# Timing of energy intake and the therapeutic potential of intermittent fasting and time-restricted eating in NAFLD

**DOI:** 10.1136/gutjnl-2023-329998

**Published:** 2023-06-07

**Authors:** Thomas Marjot, Jeremy W Tomlinson, Leanne Hodson, David W Ray

**Affiliations:** 1 Oxford Centre for Diabetes Endocrinology and Metabolism, NIHR Oxford Biomedical Research Centre, Churchill Hospital, University of Oxford, Oxford, UK; 2 Oxford Liver Unit, John Radcliffe Hospital, Oxford University Hospitals NHS Foundation Trust, Oxford, UK; 3 Sir Jules Thorn Sleep and Circadian Neuroscience Institute, University of Oxford, Oxford, UK

**Keywords:** NONALCOHOLIC STEATOHEPATITIS, ENERGY METABOLISM, LIVER METABOLISM, DIABETES MELLITUS, OBESITY

## Abstract

Non-alcoholic fatty liver disease (NAFLD) represents a major public health concern and is associated with a substantial global burden of liver-related and cardiovascular-related morbidity and mortality. High total energy intake coupled with unhealthy consumption of ultra-processed foods and saturated fats have long been regarded as major dietary drivers of NAFLD. However, there is an accumulating body of evidence demonstrating that the timing of energy intake across a the day is also an important determinant of individual risk for NAFLD and associated metabolic conditions. This review summarises the available observational and epidemiological data describing associations between eating patterns and metabolic disease, including the negative effects of irregular meal patterns, skipping breakfast and night-time eating on liver health. We suggest that that these harmful behaviours deserve greater consideration in the risk stratification and management of patients with NAFLD particularly in a 24-hour society with continuous availability of food and with up to 20% of the population now engaged in shiftwork with mistimed eating patterns. We also draw on studies reporting the liver-specific impact of Ramadan, which represents a unique real-world opportunity to explore the physiological impact of fasting. By highlighting data from preclinical and pilot human studies, we present a further biological rationale for manipulating timing of energy intake to improve metabolic health and discuss how this may be mediated through restoration of natural circadian rhythms. Lastly, we comprehensively review the landscape of human trials of intermittent fasting and time-restricted eating in metabolic disease and offer a look to the future about how these dietary strategies may benefit patients with NAFLD and non-alcoholic steatohepatitis.

**Key messages**

- Observational data show that irregular meal patterns, skipping breakfast and night-time eating are associated with an increased risk of non-alcoholic fatty liver disease (NAFLD) and related metabolic conditions.
- Distribution of total daily energy intake away from the end of the day may improve metabolic health.
- Complete fasting between dawn and dusk during Ramadan is associated with weight loss, reduced insulin resistance and improved liver biochemistry.
- Intermittent fasting protocols can lead to>5% wt loss, reduced hepatic steatosis and improved lipid profiles in patients with NAFLD and appear superior to standard dietary and weight loss advice.
- Compared with continuous energy restriction, time-restricted eating (TRE) leads to similar reductions in body weight and intra-hepatic triglyceride but may be better tolerated and is associated with greater improvements in glycaemic control.
- TRE can reprogramme circadian outputs across multiple tissues leading to synchronisation of behaviour and physiology across a 24-hour cycle.
- TRE has emerged as a promising strategy to mitigate the adverse metabolic phenotype associated with circadian misalignment induced by night-shift working.
- As the field continues to advance, it is likely that an increasing number of society consensus statements acknowledge the value of modifying timing of calorie intake as a potential strategy for the prevention and treatment of NAFLD and non-alcoholic steatohepatitis.

## Introduction

Non-alcoholic fatty liver disease (NAFLD) represents a major public health concern affecting approximately one-quarter of the global adult population and is closely associated with the epidemic of type 2 diabetes (T2D) and obesity.^1^ While the majority of individuals living with NAFLD have isolated steatosis (non-alcoholic fatty liver), a proportion will develop non-alcoholic steatohepatitis (NASH), which predisposes to cirrhosis, primary liver cancer and both liver-related and cardiovascular-related mortality.^2 3^ From a pathophysiological perspective, NAFLD is a heterogeneous condition involving the complex interplay between immune cells, inflammatory mediators and metabolic target tissues, including adipose and skeletal muscle.^4^ These multiple converging pathogenic pathways have made drug discovery challenging and as yet, no therapeutic agents for NAFLD/NASH have progressed through late-phase trials and into licensing. The mainstay of treatment therefore continues to centre on lifestyle intervention and weight loss, which has traditionally been achieved through decreasing total calorie intake and modifications to dietary macronutrient composition.^5^ This has typically focused on limiting consumption of fructose, ultra-processed foods and saturated fats and/or prescribing a balanced Mediterranean diet, which has been shown to improve liver biochemistry and hepatic steatosis.^5–7^ However, there is also accumulating evidence that the timing of energy intake across the day may play an important role in determining an individual’s risk for NAFLD, including missing breakfast, irregular meal patterns and night-time eating. These behaviours are becoming increasingly important to consider in a modern 24-hour society where there is continuous availability of food, light at night, prevalent shiftwork and disrupted sleep/wake patterns all of which promote irregular eating patterns and an extended fed period.

The link between temporal eating habits and human disease is likely to be mediated through disruption to circadian rhythms. The circadian clock network is coordinated by a central clock in the suprachiasmatic nucleus (SCN) of the hypothalamus, which communicates via neuroendocrine signals with a number of peripheral clocks, including the liver. At a cellular level, all clocks are governed by the same transcriptional–translational feedback loop involving factors, including CLOCK, BMAL1, PER and CRY, leading to periodic regulation of multiple facets of metabolism, including glucose uptake, gluconeogenesis, lipogenesis and bile acid synthesis.^8 9^ While the central pacemaker in the SCN is predominantly influenced by the light–dark cycle, the liver clock is exquisitely sensitive to feeding patterns. Indeed, the circadian phase of the liver can be directly influenced by feeding and this is independent of both the SCN and light–dark signalling.^10 11^ There is now a wealth of rodent and human data linking circadian misalignment with metabolic dysfunction,^12 13^ including feeding exclusively during the resting phase which markedly shifts the liver clock, resulting in multiple features of the metabolic syndrome.^14 15^ Collectively, these findings have led to much interest in the use of fasting and time-restricted feeding/eating as a preventative and treatment measure for metabolic conditions, including NAFLD.

In this review, we summarise observational and epidemiological studies, which establish a link between timing of food intake and NAFLD. By drawing on data from preclinical and pilot human studies, we then present the biological rationale for manipulating energy timing to help improve metabolic health and discuss how this may be mediated through restoration of natural circadian rhythms. Lastly, we review the landscape of human trials exploring intermittent fasting (IF) and time-restricted eating (TRE) in metabolic disease and offer a look to the future as to how these dietary strategies may benefit patients with NAFLD and NASH.

### Timing of food intake as a risk factor for NAFLD and metabolic dysfunction

#### Irregular eating patterns

**Figure 1:**
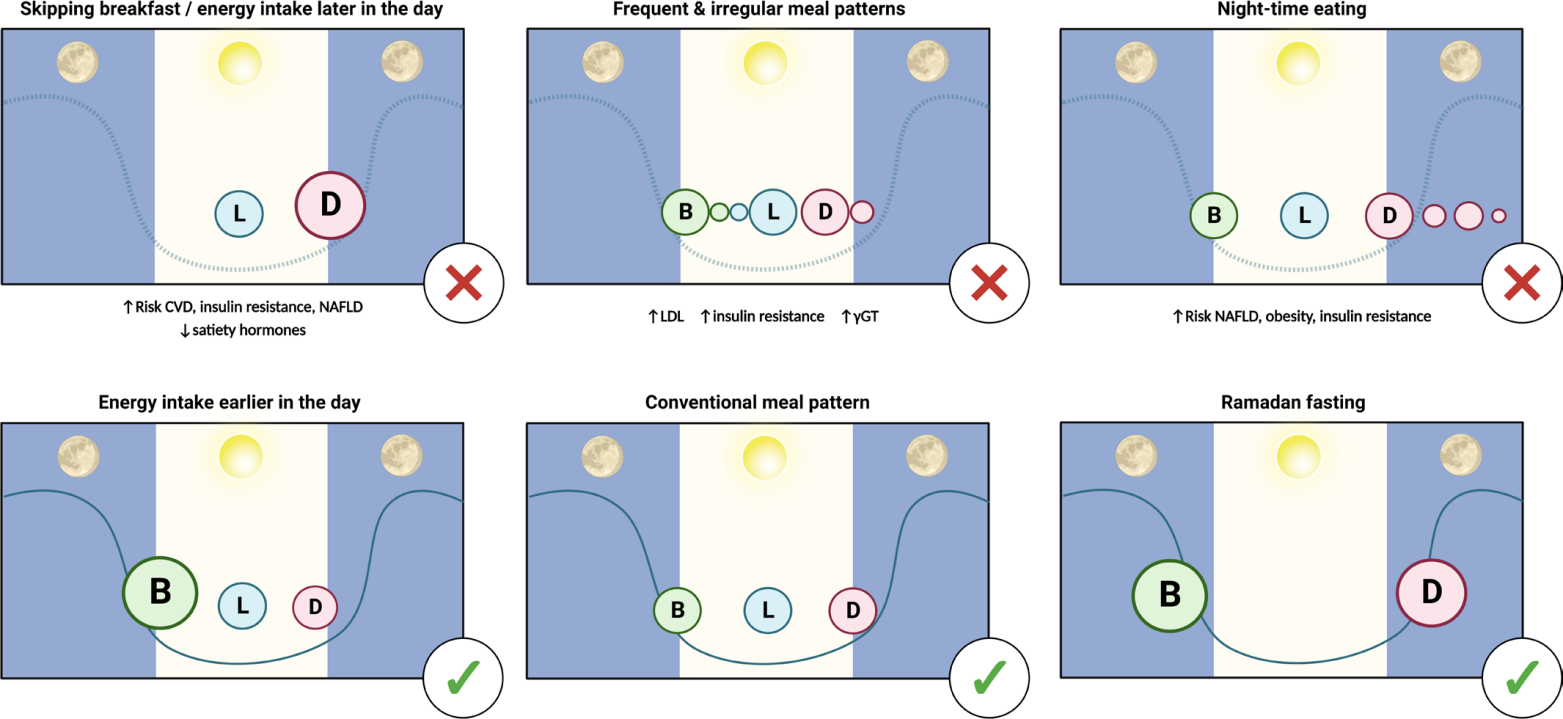
Timing of food intake as a risk factor for NAFLD and metabolic dysfunction. Timing of energy intake across the day–night cycle is an important determinant of individual risk for NAFLD. Skipping breakfast, chaotic meal patterns and night-time eating are all associated with metabolic dysfunction and disruption to natural circadian rhythms (symbolised by blue line). Conversely, shifting energy intake toward the beginning of the day, standardised meal patterns and fasting during Ramadan are all associated with benefits to metabolic and liver health. γGT, γ-glutamyl transferase; B, breakfast; CVD, cardiovascular disease; D, dinner; L, lunch; LDL, low-density lipoprotein cholesterol; NAFLD, non-alcoholic fatty liver disease. Figure made with biorender.com.

The timing of food consumption, in addition to total caloric intake and dietary macronutrient composition, is an important determinant of an individual’s predisposition to weight gain and metabolic disease (figure 1). Erratic and irregular patterns of food consumption throughout the day have been consistently shown to be deleterious.^16–18^ For example, in a population-based study of 3607 Swedish adults, irregular meal schedules (eg, eating outside of breakfast, lunch and dinner) were associated with metabolic syndrome, insulin resistance and elevated serum concentrations of γ-glutamyl transferase (γGT).^17^ Similarly, National Health and Nutrition Examination Survey data showed that eating more than 5 times per day was positively associated with body mass index (BMI) and central adiposity.^19^ These findings were corroborated in a randomised trial which showed that irregular meal patterns (variable meal frequency of 3–9 meals/day) was associated with higher post-prandial insulin concentrations, elevated low-density lipoprotein (LDL) cholesterol and insulin resistance compared with regular meal consumption (6 eating events/day).^20^ Interestingly, despite a majority of free-living individuals believing they adopt a traditional three-meal structure to calorie intake, in reality this is rarely the case.^21 22^ For example, by using smartphone image capture technology to record each time food was consumed across a 3-week period in 156 healthy adults in the USA, Gill and Panda demonstrated a distinct lack of meal clustering.^21^ Indeed, the number of discrete eating events ranged from 4 to 15 in the bottom and top deciles, respectively. Furthermore, the median interval between meals (defined as intake lasting >15 min) was 3 hours, with 25% of all meals occurring within 1.5 hours of each other and only 25% of the meals occurring after >6 hour of fasting.^21^ Similar findings were replicated in an Indian cohort where more than 50% of participants spread their caloric intake across >15 hours in a day.^22^ These non-structured eating patterns, when entrenched at a younger age, may also predict future metabolic disease with irregular eating habits in adolescents being associated with prevalent metabolic syndrome up to 25 years later.^23^

#### Skipping breakfast

The traditional mantra that breakfast is the ‘most important meal of the day’ has been the subject of much investigation. Observational studies have shown that skipping breakfast may predispose to a range of adverse health outcomes, including obesity,^24^ early onset atherosclerosis,^25^ cardiovascular disease (CVD),^26^ metabolic syndrome,^27^ insulin resistance^28^ and NAFLD.^29 30^ In a US population-based study, skipping breakfast was also associated with a higher risk of long-term cardiovascular and cerebrovascular mortality in patients with metabolic-dysfunction associated liver disease defined by hepatic steatosis on ultrasound alongside a BMI ≥23 kg/m^2^, evidence of metabolic dysregulation or T2D.^31^ Despite this, up to 30% of western populations are known to regularly omit breakfast from their daily routine.^32^ Several mechanisms have been proposed to explain the potential pathogenic role of skipping breakfast. First, the satiating properties of food are known to decline over the course of the day, and, therefore, eating breakfast may serve to minimise subsequent calorie intake and protect against weight gain.^33^ This association appears to be macronutrient specific whereby high morning carbohydrate intake is coupled with subsequent reductions in carbohydrate intake throughout the rest of the day, and likewise with protein and fat consumption.^34^ These findings are likely underpinned by distinct endocrine profiles, with satiety hormones, including peptide YY (PYY) and glucagon-like peptide 1 (GLP-1) found to be elevated during the midday period in those eating compared with those skipping breakfast.^35^ These observations are partly tempered by results of small, mostly unblinded randomised trials, which show widely inconsistent results regarding the impact of breakfast on weight and energy balance.^36^ However, post-prandial glycaemic control after lunch is known to be more robust once breakfast has been consumed.^37 38^ Using stable isotope tracer techniques, this ‘second meal phenomenon’ appears to result from insulin secretion after breakfast suppressing plasma non-esterified fatty acids (NEFA), thereby improving skeletal muscle insulin sensitivity and facilitating greater glycogen storage after lunch.^39^

Alongside these metabolic pathways, there may also be a circadian component to the benefits derived from eating breakfast.^40^ A single daily morning meal was found to advance the circadian phase in core body temperature and heart rate by 1 hour compared with a single evening meal.^41^ Similarly, a second study showed that skipping breakfast for 6 days delayed diurnal rhythms in body temperature by approximately 45 min.^42^ In addition, breakfast feeding in infants is associated with a shift in sleep–wake cycle toward an earlier chronotype with studies suggesting that morning consumption of tryptophan, the main precursor of melatonin, may improve overall sleep quality and trigger earlier sleep-onset toward the end of the day.^43 44^ Taken together, this data suggest that breakfast may act as important external cue or ‘zeitgeber’ for entrainment of circadian rhythm serving to ensure healthy synchronisation of behaviour and physiology across the day–night cycle.

#### Distribution of energy intake across the day

Beyond the benefits of simply consuming breakfast, several lines of evidence suggest that distributing total daily energy intake away from the end of the day may further improve metabolic health. For example, a Spanish population-based cohort followed up over 3.5 years demonstrated a dose–response relationship between higher percentage energy intake at lunch and lower risk of weight gain.^45^ Similarly, a cross-sectional US analysis found that consuming >33% of total energy at dinner was associated with a higher likelihood of obesity.^46^ A second Spanish cohort also examined the relationship between energy distribution and incident metabolic dysfunction. Compared with the lowest sex-specific quartile of energy intake at dinner, the OR for developing metabolic syndrome ranged from 1.71 to 2.57 between the second and fourth quartiles, respectively.^47^ This was replicated in a large cohort of 1245 non-diabetic, non-obese Italian adults followed up over 6 years, in whom a high baseline calorie intake during dinner was associated with an elevated risk of going on to develop obesity and metabolic syndrome. Lastly, further analysis in a Japanese cohort also demonstrated that a large dinner or eating just before bedtime was associated with incident NAFLD defined using non-invasive scores for steatosis, even after controlling for total daily energy intake.^48 49^ These observations may be related to the known rhythmicity of insulin sensitivity which can be up to 33% higher in the morning compared with the evening, even in healthy individuals.^50 51^ As a result, the ability to appropriately store and metabolise dietary glucose and lipid intake is progressively impaired throughout the day. Furthermore, overweight individuals randomised to consume a large breakfast were also found to have greater weight loss, lower levels of ghrelin and higher daily satiety scores compared with those receiving an isocaloric large dinner.^52^

The adverse metabolic implications of eating later in the day are further reinforced by the damaging impact of night-time eating and snacking. These behaviours have been associated with a variety of chronic disease states, including NAFLD, obesity, metabolic syndrome and CVD.^49 53 54^ Significantly higher values of plasma glucose, insulin and triglycerides are recorded after a meal eaten at night compared with an identical meal eaten during the day.^55 56^ This metabolic phenotype is reminiscent of that observed in shift-workers and jet-lagged individuals who are forced to consume food out-of-sync with the usual pattern of day and night.^57^ At the extreme end of the spectrum are patients with night eating syndrome (NES), which is characterised by evening hyperphagia, nocturnal waking for food and morning anorexia.^58^ These patients demonstrate significant changes in the timing and amplitude of various circadian markers, including eating patterns, cortisol, ghrelin and insulin, despite retaining regular sleep–wake cycling. NES, therefore, represents a good example of how eating at night can lead to dissociation between central and peripheral timing mechanisms^59^ with resultant metabolic dysfunction.^60^ Finally, circadian preference toward eveningness (late/evening chronotype) is associated with a delay in meal timing, breakfast skipping and excessive calorie intake at night,^61^ which may contribute to the higher prevalence of metabolic disease and obesity observed in this group.^62 63^ Indeed, evening chronotype may also correlate with increased severity of NASH in obese individuals, as determined by scores incorporating liver biochemistry, homeostatic model assessment for insulin resistance (HOMA-IR), waist-to-hip ratio and circulating triglycerides.^64 65^

#### Diurnal fasting: insights from Ramadan

The annual practice of fasting during Ramadan is observed by many of the >1.6 billion Muslim population worldwide and requires complete abstinence from food and drink between dawn and dusk for an entire lunar month. While calorie restriction is not mandated, intake of food does become less frequent and exclusively nocturnal. Ramadan, therefore, constitutes a major shift away from routine eating habits and represents a unique real-world opportunity to explore the physiological impact of fasting and energy distribution. The behavioural implications of Ramadan are wide ranging and heterogeneous, with effects on sleep,^66 67^ exercise^68^ and dietary composition^69^ all being reported. This has led to variable results relating to weight and metabolism across both healthy and diseased populations. Nonetheless, several meta-analyses have shown that Ramadan fasting is generally associated with weight loss,^69 70^ reduced total fat mass^70^ and improvements in cardiometabolic risk factors, including lipid profiles,^71^ blood pressure^71^ and glycaemic parameters.^72 73^ Ramadan has also been shown to improve fasting glucose and HOMA-IR specifically in patients with NAFLD in parallel with weight loss and reductions in inflammatory cytokines (eg, interleukin 6 (IL-6) and C-reactive protein (CRP)).^74^ A meta-analysis of 20 studies in predominantly healthy individuals has also identified modest but significant improvements in aspartate transaminase (AST), γGT, alkaline phosphatase and bilirubin.^75^ Several other studies have pointed towards improved markers of inflammation and oxidative stress following Ramadan fasting; however, whether these improvements occur independently of weight loss has been difficult to decipher.^76–78^

The weight loss experienced during Ramadan tends to be greatest in those who are already overweight and the majority return to their baseline weight within 5 weeks after resuming standard eating habits.^70 79^ Moreover, there is contradictory evidence regarding the effect of Ramadan on total calorie consumption with separate studies showing reduced, unchanged and increased total daily energy intake.^69^ However, pooled analysis does suggest that energy intake tends to remain consistent before and during the fasting period.^69^ The fact that weight loss and improved metabolic health often occur despite an isocaloric diet suggests that fasting may trigger protective mechanisms separate from simple energy restriction. An additional point of discrepancy is that Ramadan leads to health advantage in spite of exclusive night-time eating, which has been shown to be detrimental in other contexts as previously discussed. This shift in eating patterns is mirrored by changes in the profiles of satiety hormones, including leptin, which has an important role in the regulation of energy metabolism and food intake. Ramadan has been associated with blunting of usual nocturnal elevations in leptin as well as a delay in peak overnight concentrations, reflecting the transition to later evening meal consumption after sunset.^80 81^ However, there is currently no consistent evidence that specific hormonal profiles before, during or after dietary modification can be used as a biomarker to predict future weight regain.^82^ In addition, the eating patterns of Ramadan are associated with profound temporal alterations in other circadian biomarkers, including melatonin, cortisol, testosterone, thyroid-stimulating hormone, prolactin and insulin, which can persist up to 1 month after fasting.^83^ The sleep window is also delayed and shortened by approximately 1 hour. Therefore, despite Ramadan in many ways, exemplifying an unnatural pattern of eating, there appears to be something about the act of fasting or limiting the eating window that ultimately leads to benefit. Indeed, Ramadan fasting has been associated with upregulated key regulatory proteins involved in glucose and lipid metabolism, circadian clock function and DNA repair.^84^ This has led to much investigation regarding the pleiotropic effects of IF and TRF.

### Intermittent fasting and time-restricted eating

**Figure 2:**
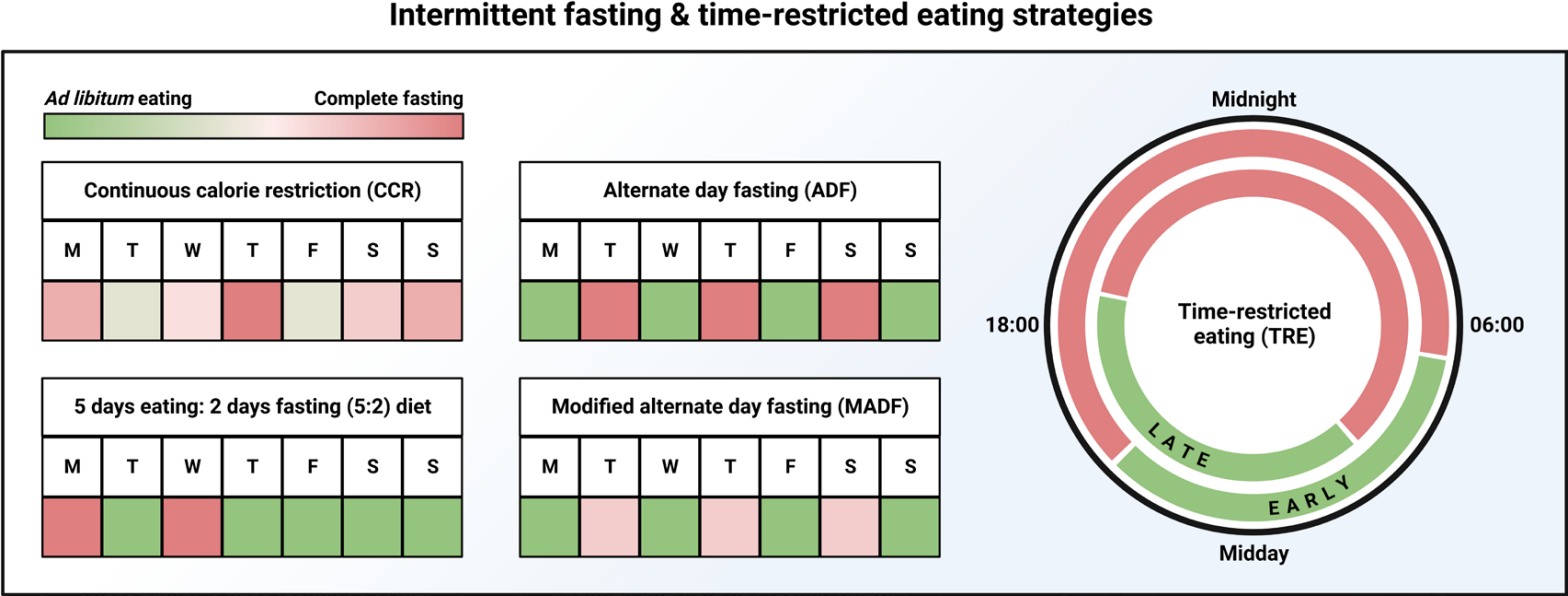
IF and TRE strategies. Commonly employed IF and TRE strategies. Figure made with biorender.com. IF, Intermittent fasting; TRE, time-restricted eating.

**Figure 3:**
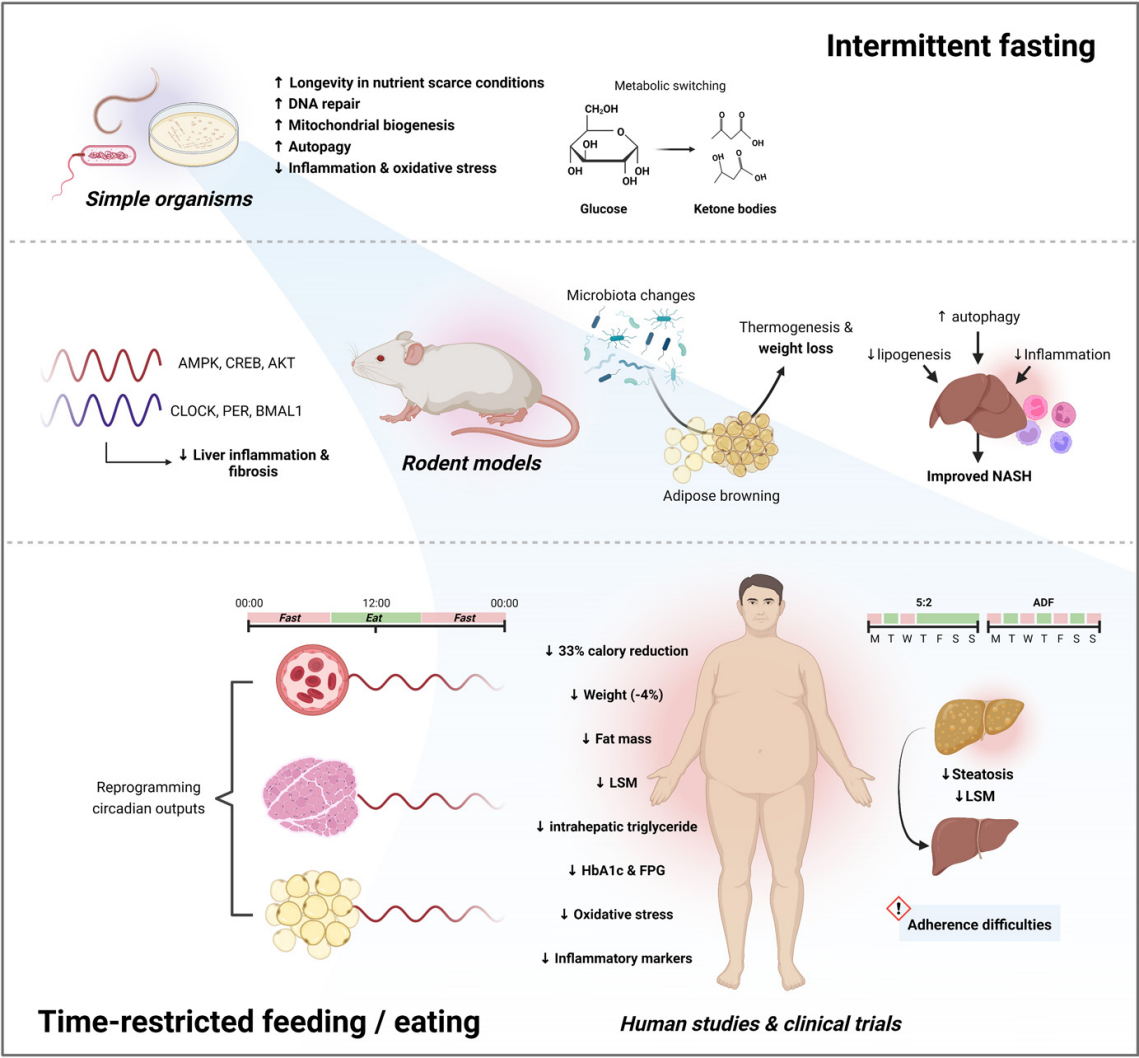
Mechanistic basis for improved metabolic outcomes following fasting and TRF/TRE. The benefits of fasting (right) and TRF/TRE (left) are well conserved across simple organisms, rodent models and humans. Fasting has pleiotropic effects, including anti-inflammatory properties, weight loss through adipose browning and exerts a beneficial impact as a result of metabolic switching from glucose to fatty acids and ketones as a source of fuel. Time-restricted calorie intake introduces additional benefits by aligning circadian rhythms across multiple tissue types. 5:2, 5 days eating 2 days fasting; ADF, alternate day fasting; FPG, fasting plasma glucose; HbA1c, glycated haemoglobin; LSM, liver stiffness measurement; NASH, non-alcoholic steatohepatitis; TRE, time-restricted eating; TRF, time-restricted feeding. Figure made with biorender.com.

In light of observational studies linking dietary patterns with chronic disease, there has been considerable interest over the last two decades in manipulating eating schedules in order to optimise metabolic health. This has revolved around two major intervention strategies: periodic fasting or IF, where caloric intake is severely constrained for short periods and TRE (or TRF in mice), which limits the daily eating window with fasting for the remainder of the day (figure 2). The two most commonly employed IF strategies in humans comprise the so-called 5:2 diet and the alternate day fasting (ADF) regimen. In the 5:2 diet, fasting occurs on 2 non-consecutive days a week with no formal energy restrictions imposed on the remaining 5 days. ADF involves a continuous alternating pattern of eating and fasting days. Modified ADF allows for some energy consumption on fasting days, usually up to 25% of normal caloric intake, whereas traditional ADF typically includes complete fasting every other day. The protective effects of fasting are well recognised across species, including simple organisms, which have evolved to avoid age-dependent damage in energy poor environments.^85^ Prokaryotes, nematodes, yeasts and mice have all been shown to have an extended lifespan when exposed to fasted or nutrient-scarce conditions^85 86^ (figure 3). The molecular mechanisms governing this response in mammals are highly conserved with coordinated reprogramming of numerous metabolic and stress resistance pathways. This includes increased DNA repair, mitochondrial biogenesis, autophagy, expression of antioxidants and downregulation of inflammatory pathways.^87^ Another fundamental adaptive response to fasting is the metabolic switch from glucose to fatty acids and ketone bodies as a source of fuel. Not only is this more energy efficient but ketone bodies also act as potent signalling molecules triggering downstream protective pathways, including those implicated in metabolic dysfunction, NAFLD and NASH (eg, via proliferator-activated receptor γ coactivator 1α and fibroblast growth factor 21).^88 89^ In addition to the positive effects of fasting, TRE may also be beneficial through entraining the circadian clock and synchronising metabolic pathways with fixed periods of feeding.^90 91^

#### Evidence for IF and TRE in preclinical models of NAFLD and NASH

Numerous studies have reported the positive effects of IF on total body weight, fat mass reduction and glucose homeostasis in rodent models^91–96^ (figure 3). For example, mice with diet-induced obesity have significant reductions in weight (~45%) when exposed to 4 weeks of ADF compared with ad libitum feeding, despite equivalent cumulative calorie intake.^95^ These changes are associated with greater insulin sensitivity, decreased hepatic triglyceride content and reduced serum alanine transaminase (ALT) concentrations. Very similar improvements in liver phenotype have also been observed following 16 weeks of a 2 day feeding and 1 day fasting (2:1) regimen in overweight mice.^94^ Adaptations within adipose tissue appear to be central to these metabolic benefits.^94 95 97^ For example, fasting has been shown to selectively stimulate beige fat development within white adipose tissue leading to increased energy expenditure and weight loss through non-shivering thermogenesis.^95^ This may be mediated through alterations to gut microbiota composition, with IF being associated with bacterial-derived elevations in acetate and lactate which subsequently drive adipose tissue browning through upregulated monocarboxylate transporter 1 expression.^95^ Furthermore, germ-free mice are resistant to fasting-induced browning, which is restored following microbiota-transplantation with subsequent improvements in metabolic homeostasis.^95^ These modifications to adipocyte physiology, however, have not clearly translated into humans in whom calorie restriction appears to improve metabolic health independent of adipose tissue browning.^98^ Fasting protocols may also directly improve liver histology in rodent models of NASH by downregulating hepatic inflammatory pathways, modifying lipogenic gene expression and increasing markers of autophagy.^96 99–102^ A single study also found that 48-hour fasting was associated with dampened hepatic stellate cell activation and reduced collagen deposition in an *Abcb4^-/-^* mouse model of chronic liver fibrosis.^103^

TRF, even with a high-fat diet (HFD), has also been shown to reverse or protect against obesity-related complications, including insulin resistance, weight gain, hepatic steatosis and systemic inflammation, in mice.^104–106^ Again, many of these changes occur independent of total calorie restriction. TRF also normalises HFD-related expression of genes involved in hepatic lipid synthesis.^106^ TRF for only 2 weeks can lead to acute reductions in hepatic lobular inflammation and endoplasmic reticulum stress, which precedes significant weight loss.^107^ In addition, in a rodent model of NASH, 6 weeks of TRF reduced histological inflammation to the same extent as combination therapy with obeticholic acid and semaglutide, with TRF also being associated with significant improvements in liver fibrosis (available as conference abstract form only).^108^ These benefits serve to highlight the physiological importance of aligning food habits, metabolic pathways and the circadian clock. Feed–fasting cycles drive daily rhythms in the activity of key regulators of nutrient homeostasis (eg, AMPK, CREB and AKT) which, in healthy conditions, run in parallel with autonomous circadian rhythms governed by clock transcription factors (eg, BMAL1, CLOCK, PER and CRY).^109^ TRF in obese mice has been shown to modify both these cyclical components by improving CREB, mTOR and AMPK pathway function and increase the expression of core clock components in the liver^104^ (figure 3). By increasing the circadian amplitude of adipocyte thermogenesis, restricting feeding during the active phase is able to protect mice from diet-induced obesity through enhanced energy expenditure.^110^ Furthermore, TRF is able to prevent the predictable evolution of obesity, metabolic syndrome, and NASH in mice with genetic or environmental clock distuption.^111 112^ These changes may be mediated by insulin and insulin-like growth factor 1 (IGF-1) production in response to food, which helps determine the phase and amplitude of circadian rhythms in vivo through increased PER synthesis.^113^ This action of insulin and IGF-1 is not restricted to any particular tissue, and facilitates widespread coordination of gene expression and behaviour with time of feeding. Lastly, although IF and TRF are often viewed separately, the mechanistic basis for these two dietary interventions are often linked and overlapping. For example, under calorie restricted conditions, mice begin to self-impose chronic cycles of 2-hour feeding and 22-hour fasting with accumulating benefits on overall lifespan.^114^ Taken together, all this evidence in rodent models points to IF and TRE as a promising treatment candidates for human metabolic disease and NAFLD.

#### Safety and efficacy of IF in patients with obesity and metabolic dysfunction

Clinical studies in healthy volunteers have shown that IF confers wide-ranging benefits on markers of metabolic health and appears safe and well tolerated even after protracted periods of up to 6 months.^115^ Despite the profound caloric restriction imposed on fasting days, participants do not appear to fully compensate by increasing caloric intake on feeding days.^115^ This accumulating energy deficit leads to weight loss and fat mass reductions. Weight independent benefits may also occur through the cardioprotective and immunomodulatory effects of circulating mediators such as ketone bodies and polyunsaturated fatty acids, which increase with fasting.^115 116^ Notably, despite considerable study heterogeneity, absolute calorie restriction (0% calories on fasting days) is rarely reported in the literature with most protocols preferring to employ 25% of daily calorie requirements on fasting days.

A number of randomised controlled trials (RCTs) have specifically explored the benefits of IF in the context of obesity and metabolic disease. These studies have consistently shown IF to be associated with an energy deficit and weight loss ranging from 3% to 8% over an intervention period of 8–12 weeks.^117^ However, in head-to-head trials, the degree of weight loss is typically equivalent to that observed with continuous calorie restriction (CCR). This is substantiated through network meta-analysis of 24 studies showing that IF regimens result in a similar degree of weight loss to CCR (mean difference: −0.26 kg over a period of 2–26 weeks).^118^ There is no clear evidence to suggest the relative superiority of either 5:2 or ADF regimens.^118^ Post-interventional increases in weight also appear comparable between IF and CCR, with one study showing that both groups regain ~30% of their accumulated weight loss within 6 months.^119^ Unlike pre-clinical studies, there is a lack of human trials which precisely match caloric intake between arms. This has made it difficult to establish whether IF contributes to weight loss and metabolic improvement independent of energy restriction. Despite variable results, IF studies in overweight patients have tended to show longitudinal improvements in glycaemic parameters including HOMA-IR, fasting glucose and insulin, although these changes are rarely superior to those observed with continuous calorie restriction (CRC).^119–121^ A single trial in patients with T2D found that both a 5:2 fasting regimen and CCR appear equally safe and effective in reducing glycated haemoglobin (HbA1c) over a 12-month period.^122^ Overall, meta-analysis of trials, including both healthy and disease populations, have shown that IF confers beneficial outcomes compared with baseline, in fasting plasma glucose, fasting insulin, HOMA-IR, as well as cardiovascular markers, including LDL-cholesterol, total cholesterol, triglycerides and blood pressure.^123^ In addition, IF may also improve systemic inflammation in overweight individuals with reduced plasma levels of amyloid A protein, IL-6, CRP,^124^ tumour necrosis factor (TNF)-α and interferon γ and increased adiponectin^120^ all being reported.

IF also appears safe with no increased frequency of gastrointestinal symptoms, fatigue, irritability or dizziness compared with standard calorie restriction.^120 125^ While there were early concerns that IF could perpetuate disordered eating behaviours (eg, binge eating, purging and negative body image perception), this has not been demonstrated in clinical trials.^125^ Caution should be exercised, however, when initiating IF or TRE in patients with T2D particularly in those on insulin or hypoglycaemic agents.^126^ IF has been associated with a small absolute increased risk of hypoglycaemic episodes, although this can be mitigated by clinician supervision, hypoglycaemia education and pre-emptive modifications to medication dosing.^127^ Adherence to IF does, however, remain challenging for some individuals with drop-out rates tending to be higher than those observed with CCR, ranging between 4% and 58%.^121 128 129^ Although the cognitive drivers of non-adherence are yet to be fully investigated, IF does require participants to precisely monitor energy intake on a regular basis which may lead to waning compliance. TRE may, therefore, be a more attractive dietary strategy for some individuals by allowing them to simply ‘watch the clock’ rather than meticulously calculate ongoing calorie consumption. This is demonstrated in meta-analysis of 24 RCTs where study retention rates used as a gross indicator of compliance were highest for TRE (94%), followed by 5:2 diet (88%) and ADF (85%).^118^

#### Clinical trials of IF in patients with NAFLD

Several studies have explored the beneficial effects of IF specifically in patients with NAFLD. Holmer *et al* performed an open-label randomised trial of a 5:2 fasting regimen compared with low-carbohydrate high-fat (LCHF) diet or standard of care (SoC) (routine lifestyle advice) in 74 patients diagnosed with NAFLD via imaging or FibroScan controlled attenuation parameter (CAP).^128^ Compared with SoC, the 5:2 and LCHF diets were both associated with greater absolute reductions in steatosis (5:2 diet: −6.1%, LCHF: −7.2%, SoC: −3.6%) and body weight (5:2 diet: −7.4 kg, LCHF −7.3 kg, SoC −2.5 kg). The 12-week 5:2 intervention also led to significant improvements in liver stiffness (−1.8 kPa), ALT (−17.6 U/L), HOMA-IR, HbA1c and LDL compared with baseline. Similarly, in a separate study, 8 weeks of ADF in patients with an established clinical diagnosis of NAFLD was associated with greater reductions in hepatic steatosis on ultrasound, improvements in hepatic sheer wave elastography and reduced ALT compared with ad libitum eating.^130^ Cai *et al* also demonstrated IF to be an effective and well-tolerated strategy for individuals with NAFLD, with ADF being associated with >5% wt loss and improvement in dyslipidaemia even after only 4 weeks.^131^ Incremental weight loss was subsequently observed at 12 weeks with concurrent reductions in fat mass superior to those randomised to control diets although there was no significant improvement in liver stiffness measurements (LSMs) compared with baseline.^131^ Notably, no trials of IF (or TRE) have been performed in overweight patients with cirrhosis. The relative risks and benefits of weight loss in this specific patient group has been subject of debate. While weight loss of 5%–10% in compensated cirrhosis has been shown to reduce liver disease progression and improve portal pressures,^132^ there have been residual concerns about the potential to precipitate sarcopenia and decompensation. Reassuringly, no decompensating events were reported in a randomised trial of semaglutide in compensated NASH cirrhosis despite 9% wt loss.^133^ Trials of IF in both elderly populations and in high-performance athletes have also not demonstrated any associations with reduced muscle mass or function.^134 135^ However, patients with decompensated cirrhosis do represent a particularly high risk group in whom weight loss interventions (including IF and TRE) should be implemented with caution alongside specialist multidisciplinary input, structured exercise and adequate protein intake in line with international societal practice guidelines.^136 137^ Lastly, the beneficial effect of night-time nutritional supplementation or an ‘evening snack’ on protein-energy balance has long been established in patients with cirrhosis, thus providing an additional reason for caution with the use of IF and TRE in this group outside of clinical trials.^138^

#### Safety and efficacy of TRE in patients with obesity and metabolic dysfunction

TRE involves limiting the window of energy intake to a specific number of hours per day with only water and zero-calorie beverages for the remainder of the day. During the eating window, individuals are usually permitted to eat ad libitum without any imposed calorie restriction. Most human trials have employed an eating window lasting between 6 hours and 10 hours. This duration has been shown to be safe, well tolerated and has tended to yield the greatest clinical benefit. Shorter windows (eg, 4 hours) are associated with an increased risk of minor adverse events (eg, headache, mood changes and nausea) without additional metabolic improvements^139^ and longer durations (≥12 hours) begin to imitate natural eating habits and have not shown any efficacy signal.^140^

Although TRE does not overtly attempt to minimise energy intake, by simply limiting the eating window to 6–10 hours, individuals naturally reduce their calorie consumption by as much as 566 kcal/day (−30%).^139^ This has consistently been associated with weight loss of approximately 3%–4% alongside corresponding reductions in total fat mass, BMI and waist circumference.^141–144^ Interestingly, however, the degree of weight loss with TRE appears to be smaller than that observed with equivalent calorie reductions achieved during standard CCR (−5% to 7%).^121 129 145^ These findings may be related to inaccurate food diary reporting in TRE trials or due to as yet unknown physiological mechanisms. Meta-analyses of RCTs has corroborated these observations showing that IF is likely to be the most effective dietary weight loss strategy, followed by CCR, and then TRE.^118^ Nonetheless, despite relatively modest weight reductions, TRE is associated with significant and often disproportionate improvements in cardiometabolic health. Indeed, several clinical studies have shown a beneficial impact on glucose regulation, oxidative stress and blood pressure independent of weight loss.^146 147^

As well as controlling the duration of eating and fasting periods, the timing of the daily eating window has been subject of increasing investigation. TRE can broadly be categorised along a spectrum between early TRE (eTRE) when the final meal is eaten mid-afternoon and late TRE (lTRE) when dinner is consumed in the evening. Several randomised trials have demonstrated superiority of early versus lTRE with regards to weight loss, glycaemic control, satiety hormone profiles (eg, reduced ghrelin and increased PYY) and hunger scores.^148–151^ Meta-analysis of studies has also indicated that eTRE is associated with the greatest improvements in insulin sensitivity.^152^ While most studies stipulate the period of energy consumption, some groups have allowed self-selection of the eating window with comparable improvements in weight and metabolic parameters.^144^ When participants are allowed to choose their preferred period of eating, the majority will select an early or intermediate timeframe where all eating is completed >2 hours before going to bed which is associated with improvements in fasting glucose.^153^ This re-enforces observational data showing that minimising energy intake in the evening is associated with improved metabolic health. Eating earlier in the day ensures that calorie intake occurs at the time of maximal insulin sensitivity, and also at the time at which the internal circadian clock is most sensitive to external inputs.

#### TRE can reprogramme circadian regulation of human metabolism

Several human studies have directly explored the impact of TRE on circadian markers. First, TRE has been associated with an increase in the amplitude of cortisol rhythms while melatonin levels, which are predominantly influenced through light–dark signalling, remain unaffected.^154^ Detailed metabolic phenotyping of obese male participants has also demonstrated marked alterations in 24-hour profiles of insulin, NEFA and triglycerides following 8 weeks of self-selected 10-hour TRE.^154^ These systemic changes seem to occur rapidly after dietary intervention, with a second study in overweight individuals showing similar shifts in circulating insulin, NEFA, gastric inhibitory peptide and GLP-1 after only 4 days of TRE.^155^ These fluctuations occur in parallel with increased whole blood expression of multiple clock genes and genes involved in longevity and autophagy (eg, SIRT1 and BDNF).^148^ These findings were corroborated in a further study in healthy volunteers, which also showed widespread enhancement of clock gene expression in peripheral blood, which was more pronounced with early compared with mid-day TRE.^156^ In addition to circulating biomarkers, TRE also has a direct impact on the rhythmic behaviour of metabolically active tissues known to be integral to the pathogenesis of NAFLD, including adipose and skeletal muscle. In subcutaneous adipose tissue, TRE has the remarkable ability to trigger the oscillation of hundreds of genes which are otherwise arrhythmic at baseline.^154^ This includes genes involved in adipogenesis, adipose browning and pathways mediating lipogenesis (eg, SREBF1) which has an important role in the sensitisation of adipose tissue to insulin.^157^ Similarly, short-term TRE in overweight men tended to advance the circadian phase of both circulating serum metabolites and genes controlling amino acid transport in skeletal muscle.^158^ These latter changes, however, occurred in the absence of alterations to muscle clock gene oscillations, suggesting that TRE may have tissue-specific effects on metabolic function independent of the autonomous clock machinery. Further experimental work in humans is required to decipher the role of feeding cues in reprogramming diurnal metabolism, including the differential impact on central and peripheral clocks. Several studies have also shown beneficial effects of TRE on sleep quality and duration further implicating the role of eating patterns in establishing circadian realignment.^159^ This concept is also demonstrated by accumulating data showing a benefit of TRE in shift-workers with circadian misalignment who tend to have longer eating windows and higher total energy intake.^160^ In a recent randomised trial, including 24-hour shift-working firefighters, TRE improved very-low-density lipoprotein (VLDL) particle size, quality of life and sleep disturbance, and led to reductions in HbA1c and blood pressure in those with elevated cardiovascular risk at baseline.^161^

#### Therapeutic potential of TRE in NAFLD

**Figure 4:**
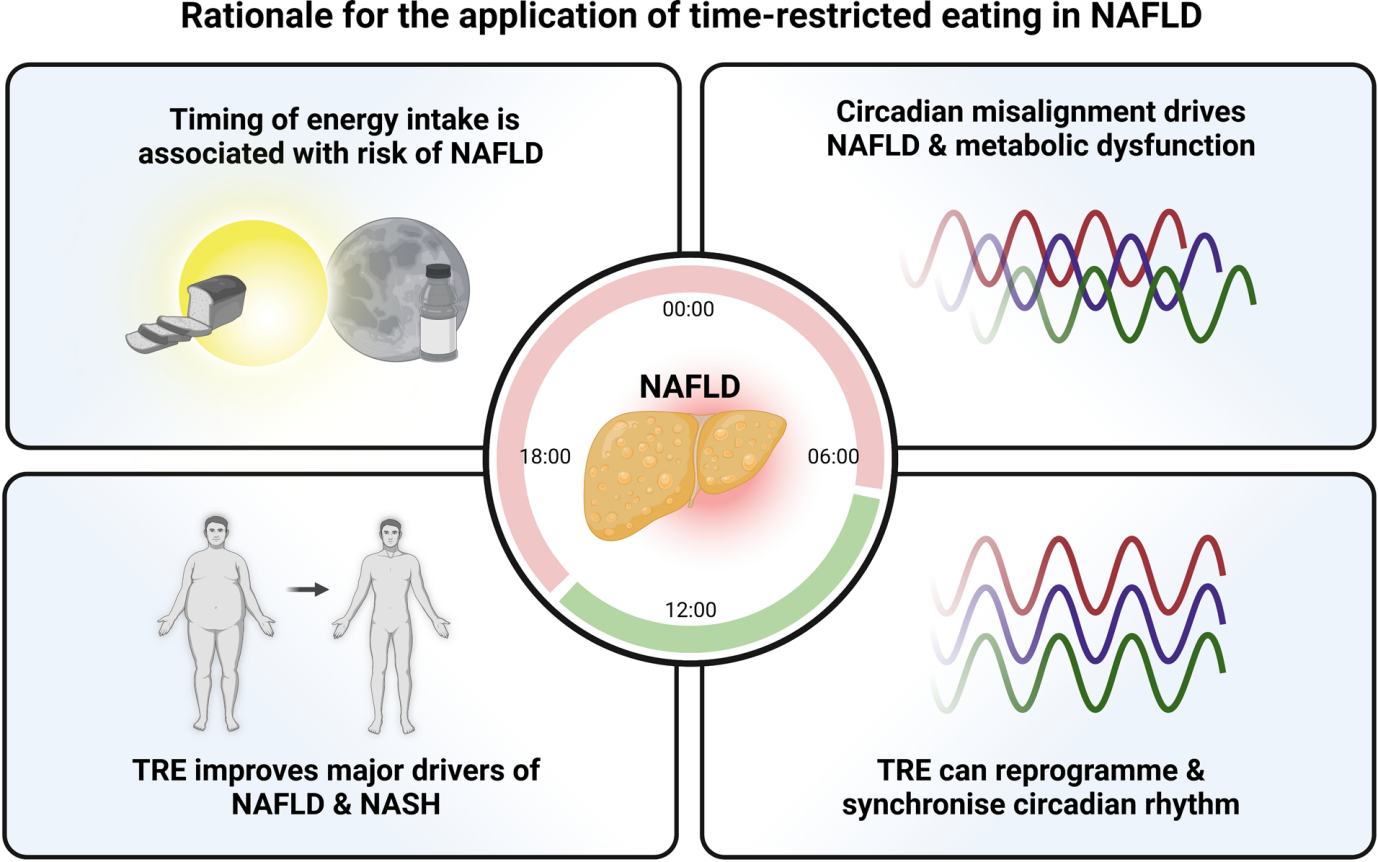
Rationale for the application of TRE in NAFLD. Multiple strands of evidence point toward the therapeutic potential of TRE in NAFLD and NASH. Timing of energy intake, circadian rhythms and metabolic phenotype are all intrinsically linked. These interactions can be positively influenced by TRE which has been shown to restore the rhythmicity of metabolic pathways and is known to improve major drivers of liver disease progression, including obesity and insulin resistance. NAFLD, non-alcoholic fatty liver disease; NASH, non-alcoholic steatohepatitis; TRE, time-restricted eating. Figure made with biorender.com.

TRE research over the last two decades has largely revolved around single-arm, proof-of-concept longitudinal studies or small pilot trials over short durations with improvements in weight and cardiovascular markers as the most common primary outcomes. Nonetheless, the wide reproducibility of positive findings has allowed the field to expand exponentially with numerous large, more rigorous RCTs planned or underway across an expanding range of chronic health conditions.^144^ There is now clear rationale for further exploration of TRE specifically in NAFLD. The background evidence is compelling as discussed above; circadian misalignment is associated with hepatic metabolic dysfunction, chaotic dietary patterns predispose to steatosis and inflammation, TRE can reprogramme circadian outputs and has been shown to improve key drivers of NAFLD progression, including weight and insulin resistance (figure 4).

Some data do currently exist which report a benefit of TRE in patients with NAFLD. In a Chinese trial, Cai *et al* reported outcomes in 96 NAFLD patients with high LSM at baseline (>9.6 kPa) who were randomised to a self-selected 8 hour TRE window.^131^ Compared with control diet, TRE was well tolerated and associated with significant weight loss (−4.8%), decreased fat mass and improved triglycerides over 12 weeks. However, there was no change in LSM likely due to the relatively short intervention period and the study did not include assessments of steatosis, inflammation, liver biochemistry or circadian markers at baseline or follow-up. A second RCT in T2D showed that eating breakfast and lunch (between 06:00 and 16:00) over 12 weeks was more effective at reducing body weight, fasting plasma glucose and intrahepatic triglyceride (IHTG) than the same calorie restriction split across six small meals throughout the day. Lastly, a rigorous trial by Wei *et al* randomised 88 patients with obesity and NAFLD to receive either TRE with calorie restriction (08:00 and 16:00) or calorie restriction alone for 12 months. All participants consumed <1800 kcal/day for men and <1500 kcal/day for women and received regular telephone reminders and face-to-face dietician input. Both interventions resulted in substantial weight loss of >10% with marked reductions in both IHTG and LSM, emphasising the importance of achieving energy deficit in the management of obesity and NAFLD. While TRE significantly improved HOMA-IR compared with daily calorie restriction, it was not superior in reducing IHTG as the primary endpoint. This may be related to the study’s particularly stringent calorie targets masking relatively subtle benefits derived from differential food timing. Exploring whether TRE can achieve meaningful clinical benefits with less aggressive calorie restriction will help establish whether this strategy has wider real-world applicability outside of a clinical trial setting.

Two further RCTs in individuals without established NAFLD have reported a tendency towards improved liver biochemistry with TRE. Xie *et al* showed a modest but significant reduction in plasma AST in healthy volunteers randomised to eTRE (−3 U/L) compared with control diet, which occurred in parallel with reduced inflammatory mediators (eg, TNF-α and IL-8), improved insulin sensitivity, upregulated clock gene expression and enhanced gut microbial diversity.^156^ A second study of 10-hour TRE for 12 weeks in patients with metabolic syndrome was also associated with a 10% reduction in both AST and ALT, although this did not reach statistical significance (p=0.09).^147^ Further dedicated trials incorporating liver-specific outcomes are required in well-phenotyped patients with NAFLD/NASH in order to establish efficacy and decipher underlying mechanistic pathways contributing to improved liver and metabolic health. Several additional RCTs of TRE in patients with NAFLD are currently registered with clinicaltrials.gov. These report a range of target sample sizes reaching a maximum of 400 patients (NCT05579158) and have variable intervention time-frames between 6 weeks and 16 weeks. Primary outcomes in these active trials have predominantly focused on hepatic steatosis assessed by magnetic resonance techniques or CAP. Some groups also plan to report on hepatic de novo lipogenesis (NCT04997486) as well as markers of fibrogenesis, oxidative stress and changes in gut microbiota (NCT05220956). It remains unclear to what extent these trials will explore the effect of TRE on circadian rhythms, sleep, and diurnal patterns of liver metabolism and inflammation. Future studies will need to establish the longer-term efficacy and tolerability of these regimens in patients with NAFLD and directly compare different fasting regimens and calorie restriction thresholds.

Lastly, despite accumulating human data signalling the benefits of IF and TRE, these dietary interventions are currently under-represented in national and international clinical practice guidelines relating to metabolic disease, including NAFLD (table 1). This is likely to be partly related to the lack of currently available data from well-powered trials reporting on clinical outcomes or robust surrogate endpoints. There is also little acknowledgement in clinical guidelines that unhealthy timing of energy intake (eg, skipping breakfast and night-time eating) may represent discreet risk factors for metabolic disease. In the coming years, as the field continues to advance, it is likely that an increasing number of consensus statements acknowledge the value of modifying timing of calorie intake as a potential strategy for NAFLD prevention and treatment.

**Table 1:** Reference to timing of energy intake in societal guidelines for NAFLD and metabolic disease

Society clinical practice guideline	Patient group	Year	Dietary recommendations	Specific reference to timing of energy intake	Specific reference to IF and/or TRE
EASL—EASD—EASO^162^	NAFLD	2016	∙ Energy restriction ∙ Reduce processed and high-fructose foods ∙ Mediterranean diet	–	–
AASLD^163^	NAFLD	2022	∙ Prescribe a diet leading to calorie deficit ∙ Encourage Mediterranean diet	–	IF included as potential dietary strategy to achieve calorie deficit
AGA^164^	NAFLD	2021	∙ Hypocaloric diet ∙ Should follow the Mediterranean diet	–	∙ Potential benefits of IF and TRE but require an individualised approach before prescribing ∙ Additional studies are needed before IF and TRE can routinely be considered
APASL^165^	MAFLD	2020	∙ Energy restriction ∙ Reduce processed and high-fructose foods ∙ Mediterranean diet	–	–
BASL and BSG^166^	NAFLD	2022	∙ Tailored dietary advice to achieve a calorie deficit ∙ Reduction of refined carbohydrates and processed foods ∙ Increased consumption of vegetables, lean protein and fish	–	–
EASO^167^	Obesity	2015	∙ Individualised energy restriction diets ∙ Decrease energy density of foods and reduce ∙ Portion sizes ∙ Manage and reduce episodes of loss of control or binge eating	∙ Avoid snacking between meals ∙ Do not skip breakfast ∙ Avoid eating at night	–
ACC—AHA—The Obesity Society^168^	Obesity	2013	∙ Prescribe an individualised diet leading to calorie deficit (eg, low-carbohydrate, low-protein, Mediterranean, low-glycaemic load)	–	–
EASD and ADA^168^	T2D	2022	∙ Individually selected diet to achieve energy deficit ∙ Mediterranean and low carbohydrate diet	–	∙ IF likely to lead to similar improvements in glycaemic control to CCR ∙ Caution with insulin and sulphonylureas
ADA^169^	Obesity in T2D	2022	∙ Individualised nutritional recommendations to achieve energy deficit	–	–

AASLD, American Associate for the Study of the Liver Diseases; ACC, American College of Cardiology; ADA, American Diabetes Association; AGA, American Gastroenterology Association; AHA, American Heart Association; APASL, Asian Pacific Association for the Study of the Liver; BASL, British Association for the Study of the Liver; BSG, British Society for Gastroenterology; CCR, continuous calorie restriction; EASD, European Association for the Study of Diabetes; EASL, European Association for the Study of the Liver; EASO, European Association for the Study of Obesity; IF, intermittent fasting; MAFLD, metabolic-dysfunction associated fatty liver disease; NAFLD, non-alcoholic fatty liver disease; T2D, type 2 diabetes; TRE, time-restricted eating.

## Conclusion

Temporal eating patterns, liver homeostasis, circadian clock function and metabolic health are all intrinsically linked. Food intake is an extremely powerful external timing cue, which serves to synchronise rhythms of hepatic energy metabolism to fed-fasted cycles, independently of the light–dark cycle. As a result, certain non-physiological eating behaviours, including missing breakfast, irregular meal patterns and night-time snacking, are closely associated with an adverse metabolic and liver phenotype. However, this has presented an exciting opportunity for therapeutic intervention though manipulating the timing of calorie intake. IF protocols in humans have drawn on a wealth of preclinical data showing that multiple advantageous metabolic pathways are upregulated in nutrient scarce conditions. Furthermore, TRE may facilitate incremental benefits beyond fasting by resynchronising circadian rhythms across multiple metabolically active tissues involved in the pathogenesis of NAFLD. As a result, TRE has been shown in clinical trials to be a safe and effective way to reduce steatosis and improve multiple domains of the metabolic syndrome. Taken together, there is a strong rationale for further detailed exploration of the liver-specific benefits of TRE and this dietary strategy may emerge as a promising management option for patients with NAFLD and NASH.
